# Supplementary material for: Programming bacteria for multiplexed DNA detection
Source: Nat Commun. 2023 Apr 10;14:2001. doi: 10.1038/s41467-023-37582-x (PMC10086068; doi:10.1038/s41467-023-37582-x)
Supplement: Supplementary file 1 — Supplementary Information [file 41467_2023_37582_MOESM1_ESM.pdf]

Supplementary Information

Programming bacteria for multiplexed DNA detection

Yu-Yu Cheng[1+], Zhengyi Chen[1+], Xinyun Cao[1], Tyler D. Ross[1],
Tanya G. Falbel[2], Briana M. Burton[2], and Ophelia S. Venturelli[1,2,3*]

[1]Department of Biochemistry, University of Wisconsin - Madison, WI, United States
[2]Department of Bacteriology, University of Wisconsin - Madison, WI, United States
[3]Department of Chemical & Biological Engineering, University of Wisconsin-Madison

[+]These authors contributed equally to this work: Yu-Yu Cheng, Zhengyi Chen.
[*]To whom correspondence should be addressed: venturelli@wisc.edu

This file includes:

Supplementary Figure 1. Plasmid maps for the construction of DNA-sensing *B. subtilis*.
Supplementary Figure 2. Sequencing of transformed *E. coli* sensor and escape mutants.
Supplementary Figure 3. Nucleotide BLAST search of target sequences in EC and ST sensors.
Supplementary Figure 4. GFP expression of ST, SA, and CD sensors in selective liquid medium.
Supplementary Figure 5. Growth curve of DNA sensors in selective liquid medium.
Supplementary Figure 6. Orthogonality test of the four constructed DNA sensors.
Supplementary Figure 7. Transformation efficiency based on transformed sensor colony number.
Supplementary Figure 8. Fluorescence images of transformed EC-G, ST-R, and SA-B sensors.
Supplementary Figure 9. Multiplexed detection in complex DNA samples without target species.
Supplementary Figure 10. Detection of spike-in *E. coli* or *S. typhimurium* in mouse ceca.
Supplementary Table 1. List of plasmids.
Supplementary Table 2. List of bacterial strains.
Supplementary Table 3. Sequences of genetic parts.
Supplementary Table 4. Sequences of primers.

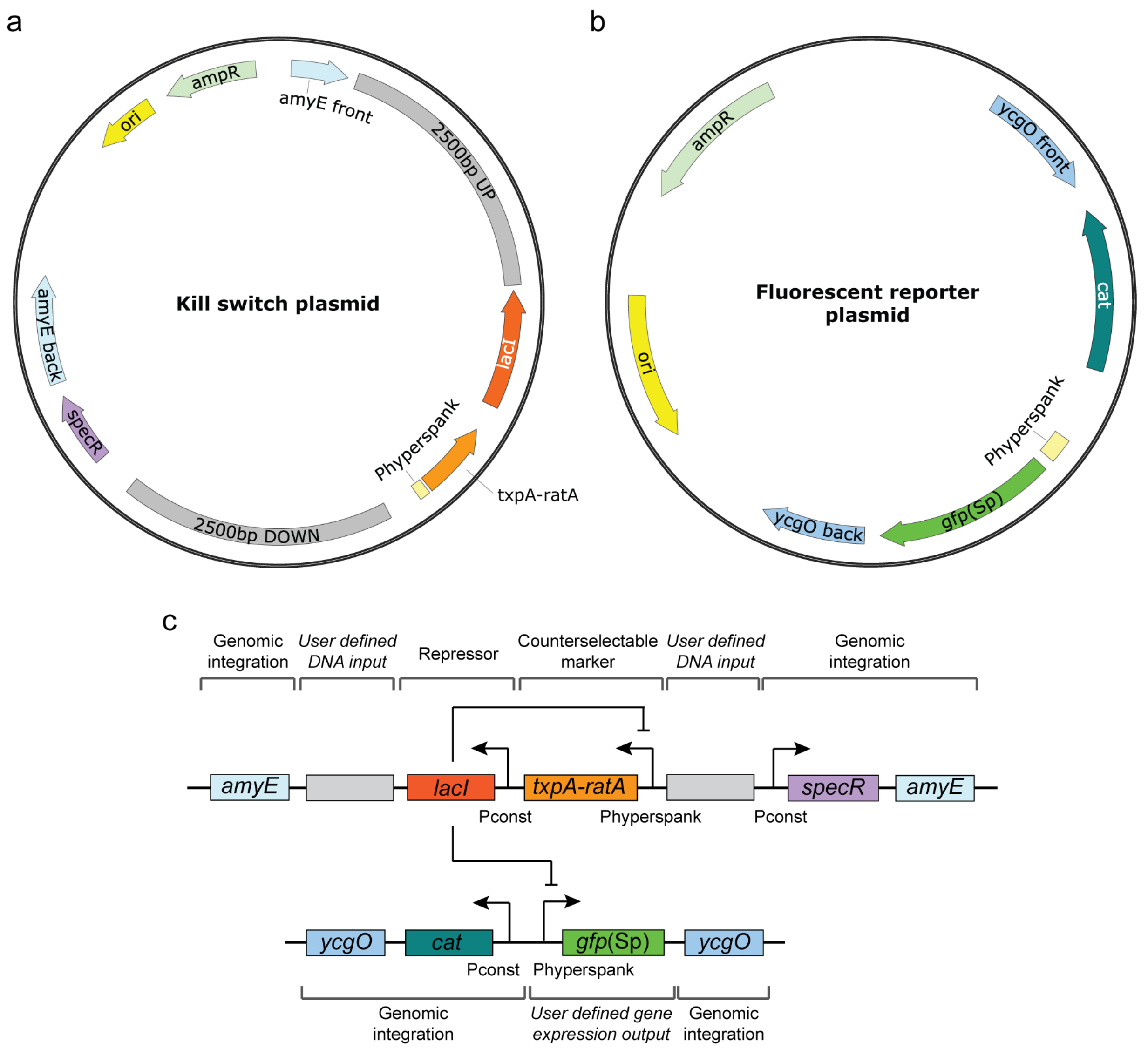

Supplementary Figure 1. Plasmid maps for the construction of DNA-sensing *B. subtilis*. (a) The kill switch plasmid constructed for the DNA detection via homologous recombination. Repressor LacI regulates the expression of the toxin-antitoxin system *txpA-ratA.* The landing pads upstream and downstream of *lacI* and *txpA-ratA* can be introduced with target DNA sequences. The plasmid can be integrated into the *amyE* locus on *B. subtilis* PY79 genome by spectinomycin selection. **(b)** The fluorescent reporter plasmid constructed for the DNA detection. The green fluorescent protein GFP(Sp) is regulated by the repressor LacI. The plasmid can be integrated into the *ycgO* locus on *B. subtilis* PY79 genome by chloramphenicol selection. The green fluorescent protein *gfp*(Sp) was codon-optimized for *Streptococcus pneumoniae* and displayed high fluorescence signal in *B. subtilis*[1]. **(c)** Modular synthetic genetic circuit design allows customized input target DNA sequence and output gene expression.

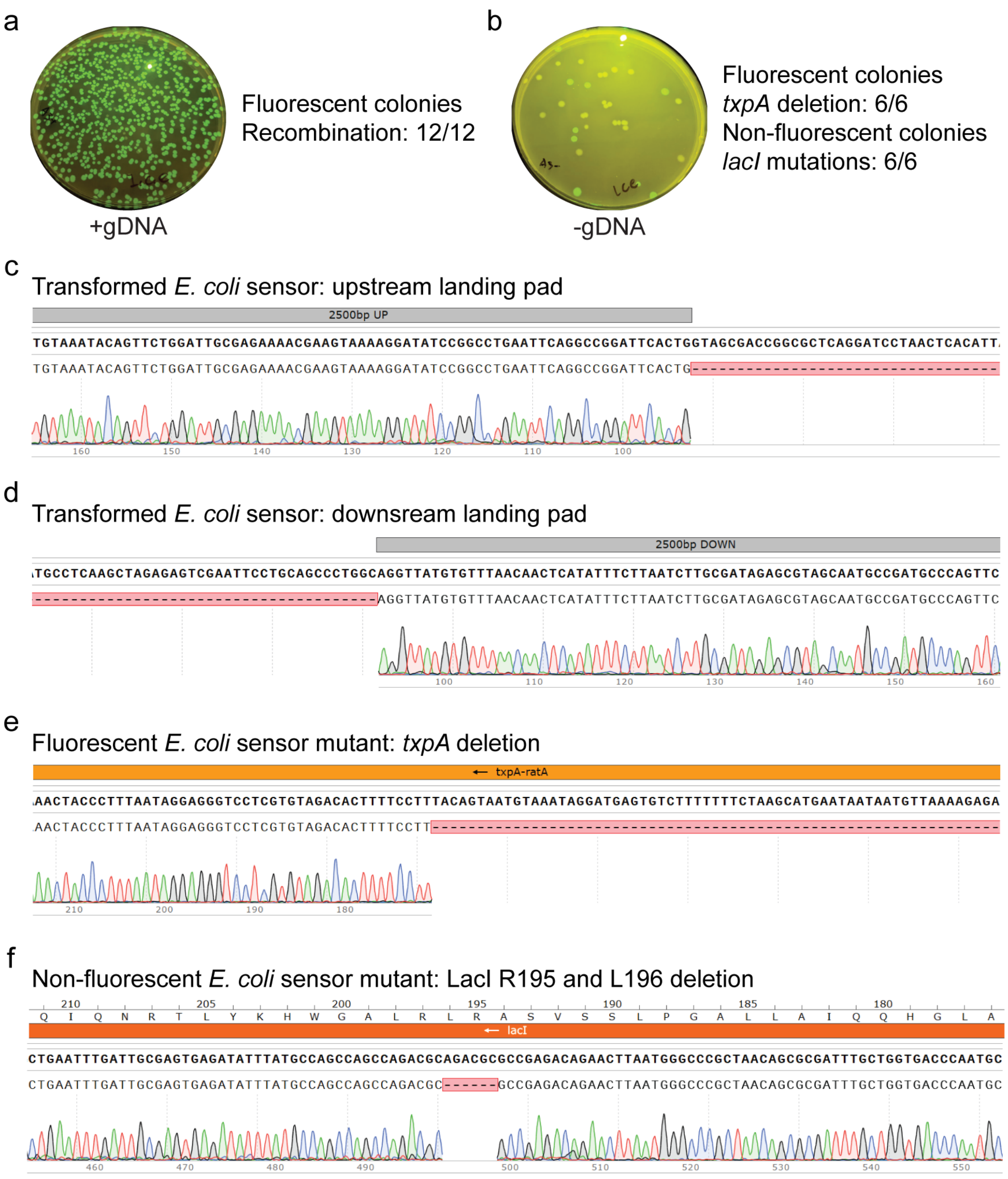

Supplementary Figure 2. Sequencing of transformed *E. coli* sensor and escape mutants. Fluorescence images of *E. coli* DNA sensor colonies on selective agar plates after being transformed with **(a)** 100 ng/mL purified *E. coli* gDNA or **(b)** no DNA. **(c)(d)** gDNA of transformed *E. coli* sensor was sequenced by Sanger sequencing to confirm the homologous recombination that joined the two landing pads and removed the repressor *lacI* and toxin-antitoxin system *txpA-ratA* (12/12 colonies with joint landing pads). **(e)** A GFP-expressing escape mutant had deletion in the toxin *txpA*. Mutants with a non-functional toxin can grow and express GFP in the presence of IPTG (6/6 colonies with different mutations in *txpA*). **(f)** A non-fluorescent escape mutant had deletion of R195 and L196 in the repressor LacI, which could affect IPTG binding[2]. Mutants with a non-functional LacI can grow in the presence of IPTG without fluorescence expression (6/6 colonies with different mutations in *lacI*).

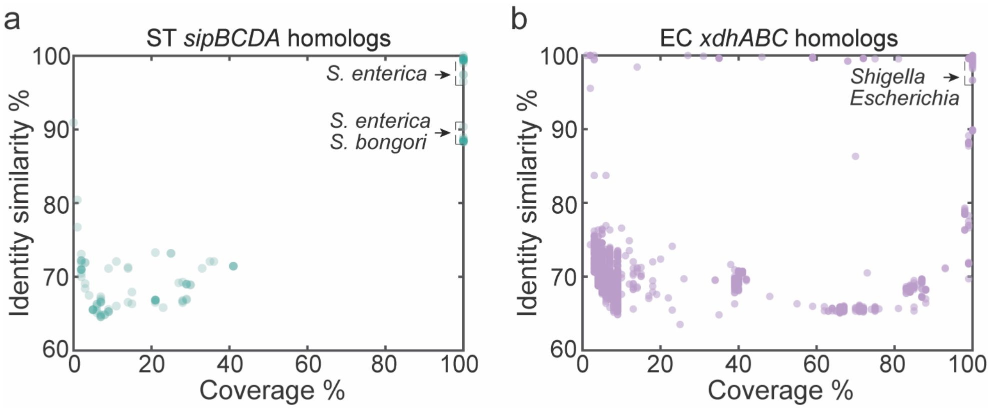

Supplementary Figure 3. Nucleotide BLAST search of target sequences in EC and ST sensors. (a) Nucleotide BLAST search of 5000 bp *S. typhimurium sipBCDA* in the NCBI database. Each circle represents a homolog with specific coverage and identity similarity found in species different from *S. typhimurium*. Homologs were found mostly in the *Salmonella enterica* (*S. enterica*) species but rarely found in other species. **(b)** Nucleotide BLAST search of 5000 bp *E. coli* MG1655 *xdhABC* in the NCBI database. Each circle represents a homolog with specific coverage and identity similarity found in species different from *E. coli*. Highly similar homologs were found in the closely related *Shigella* and *Escherichia* species. The region within dashed lines are non-target species that could be recognized by the sensor based on the high conservation of the target sequence.

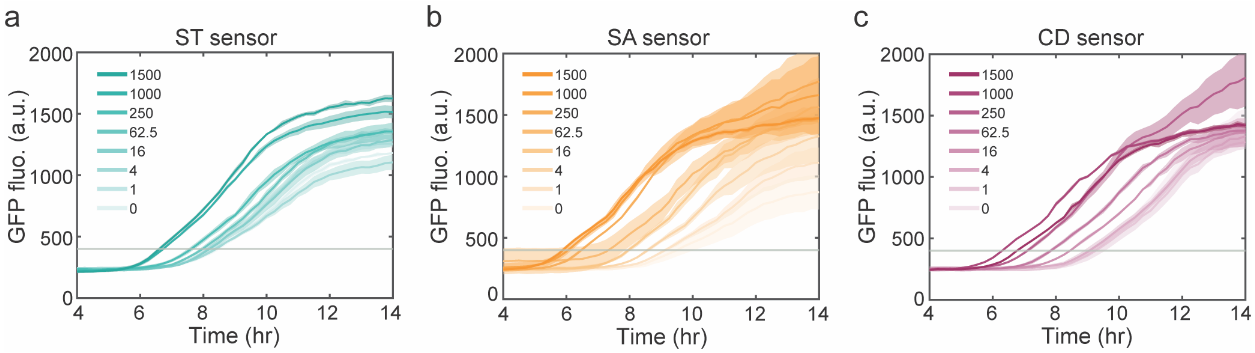

Supplementary Figure 4. GFP expression of ST, SA, and CD sensors in selective liquid medium. Time-series measurements of GFP expression of **(a)** ST sensor, **(b)** SA sensor, and **(c)** CD sensor in liquid medium after being transformed with varying target species' gDNA concentrations (ng/mL). A threshold of GFP fluorescence 400 was used to determine the detection time for each gDNA concentration. GFP expression of sensors in LB medium supplemented with 2 mM IPTG correlated with the gDNA concentration supplemented for transformation. Line is the average of four technical replicates and the shaded region represents one standard deviation from the average.

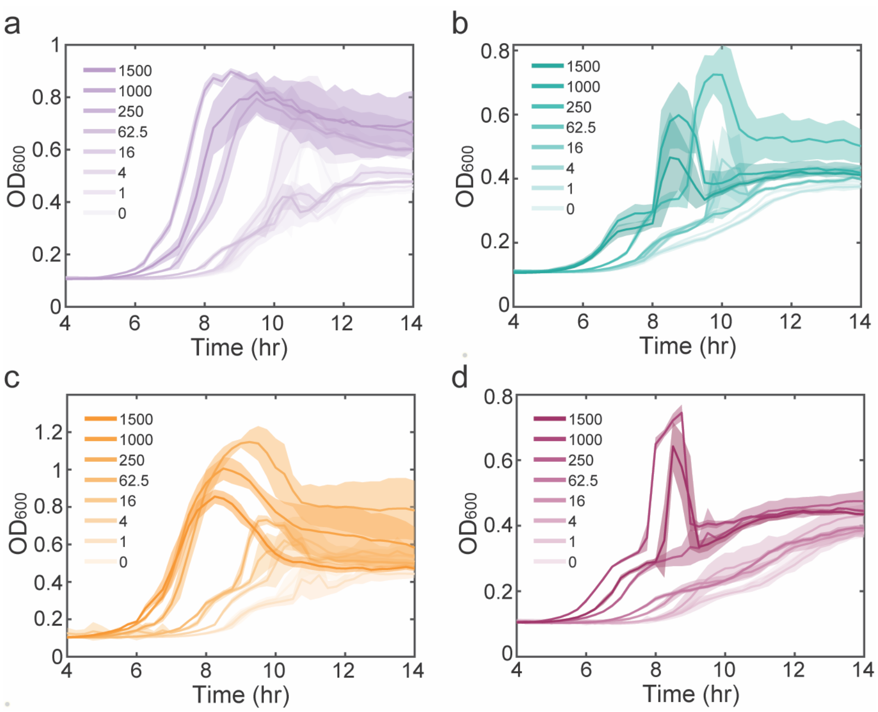

Supplementary Figure 5. Growth curve of DNA sensors in selective liquid medium. Time-series measurements of OD600 absorbance of **(a)** EC sensor, **(b)** ST sensor, **(c)** SA sensor, and **(d)** CD sensor in liquid medium after being transformed with varying target species' gDNA concentrations (ng/mL). Sensor growth in LB medium supplemented with 2 mM IPTG correlated with the gDNA concentration supplemented for transformation. Line is the average of four technical replicates and the shaded region represents one standard deviation from the average.

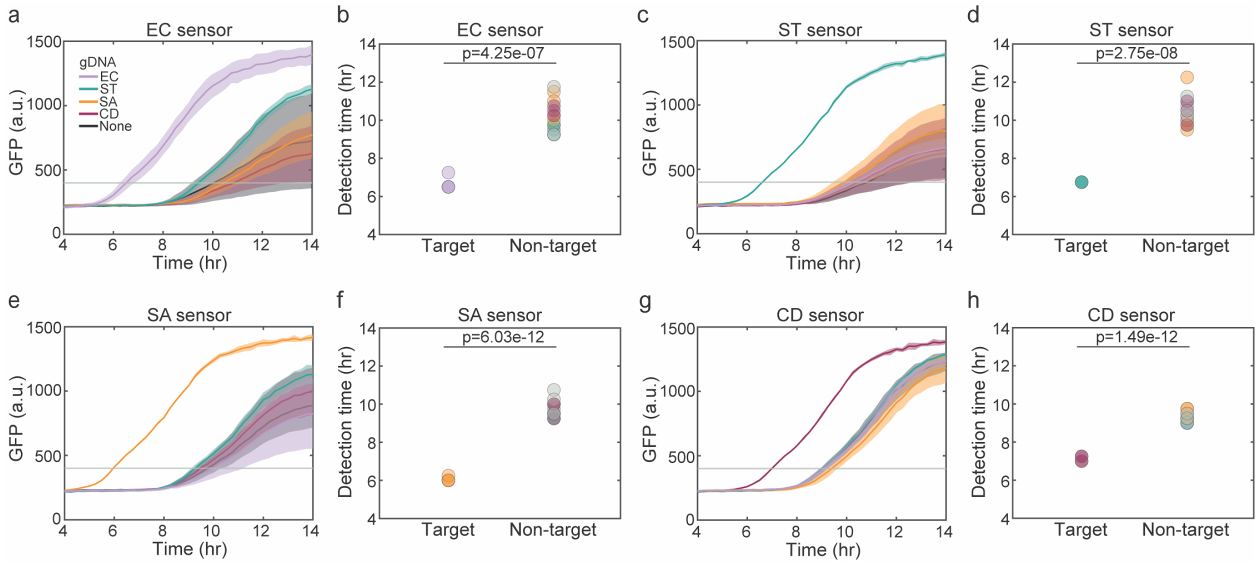

Supplementary Figure 6. Orthogonality test of the four constructed DNA sensors. Time-series measurements of GFP expression of **(a)** EC sensor, **(c)** ST sensor, **(e)** SA sensor, and **(g)** CD sensor in selective liquid medium after being transformed with gDNA extracted from different

strains or no gDNA. A threshold of GFP 400 was used to determine the detection time for the target gDNA and non-target gDNA or no gDNA for **(b)** EC sensor, **(d)** ST sensor, **(f)** SA sensor, and **(h)** CD sensor. Unpaired *t*-test was performed to determine if the detection time for the target gDNA was different from non-target gDNA and no gDNA. Line is the average of four technical replicates and the shaded region represents one standard deviation from the average.

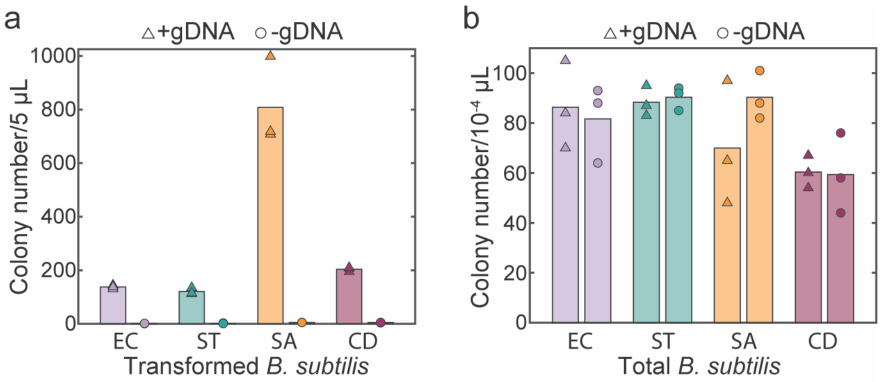

Supplementary Figure 7. Transformation efficiency based on transformed sensor colony number. (a) Transformed sensor colony number per 5 μL after being transformed with 100 ng/mL target gDNA or no DNA. **(b)** Total sensor colony number per 10^{-4} after being transformed with 100 ng/mL target gDNA or no DNA. The transformation efficiency in **Fig. 2a** was calculated by the ratio of the density of transformed sensor to the density of total sensor. Bar represents the average of three biological replicates.

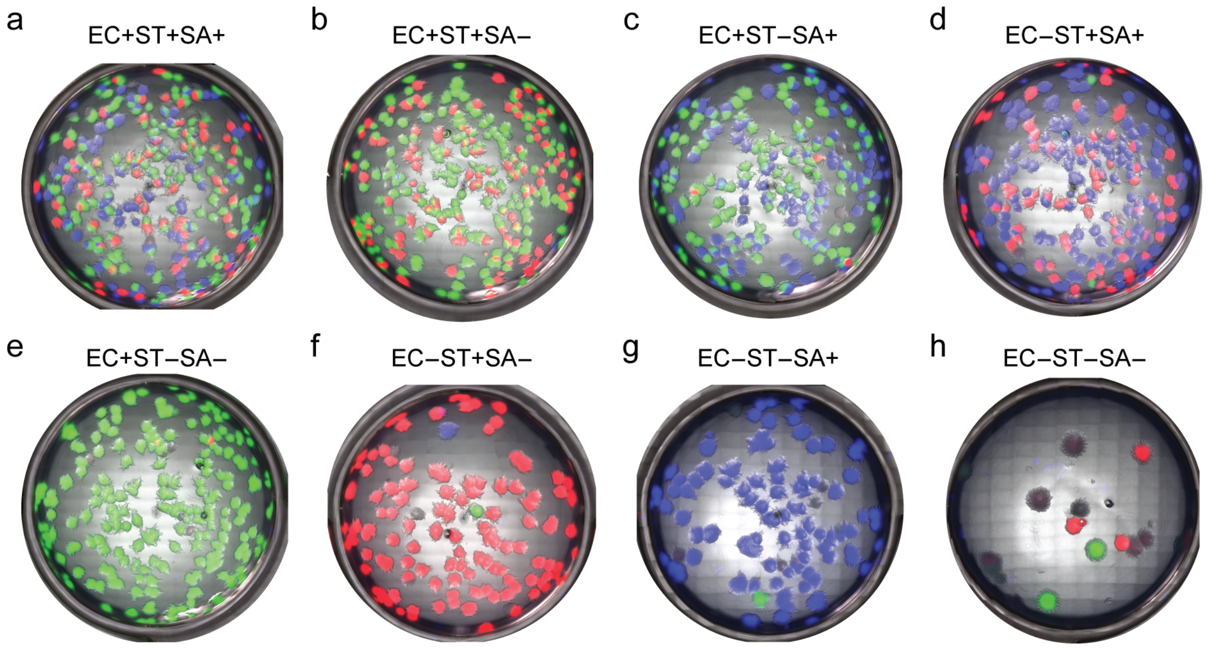

Supplementary Figure 8. Fluorescence images of transformed EC-G, ST-R, and SA-B sensors. GFP, RFP, or BFP-expressing colonies on selective agar plates after the mixed culture

of EC-G, ST-R, and SA-G sensors were transformed with different combinations of gDNA (200 ng/mL): **(a)** *E. coli*, *S. aureus* and *S. typhimurium* gDNA, **(b)** *E. coli* and *S. typhimurium* gDNA, **(c)** *E. coli* and *S. aureus* gDNA, **(d)** *S. typhimurium* and *S. aureus* gDNA, **(e)** *E. coli* gDNA, **(f)** *S. typhimurium* gDNA, **(g)** *S. aureus* gDNA, and **(h)** no gDNA.

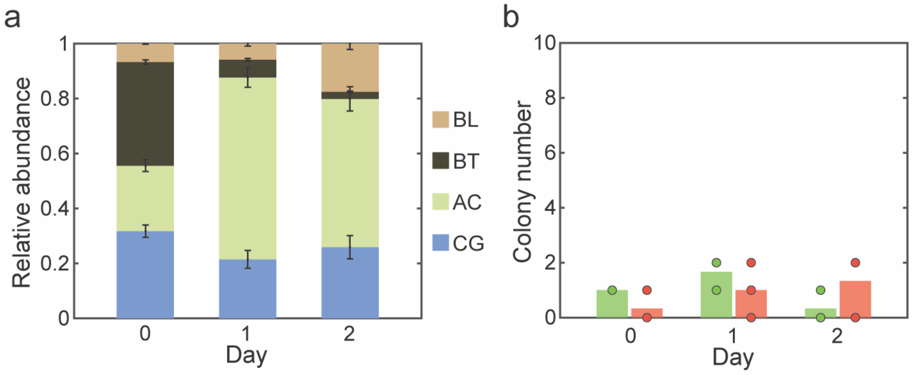

Supplementary Figure 9. Multiplexed detection in complex DNA samples without target species. (a) Relative abundance of four species in a synthetic gut microbial community composed of *B. longum* (BL), *B. thetaiotaomicron* (BT), *A. caccae* (AC), and *C. asparagiforme* (CG). The four bacteria were co-cultured in YBHI medium anaerobically for 2 days. 16S rRNA gene of each strain was PCR amplified and sequenced by NGS to determine their relative abundance at different days. Bar represents the average of three technical replicates of 16S rRNA gene sequencing with one standard deviation as the error bar. **(b)** Numbers of GFP or RFP-expressing colonies of SA-G and ST-R sensors on selective agar plates after being transformed with gDNA extracted from the microbial community without the target strains. Bar represents the average of three technical replicates.

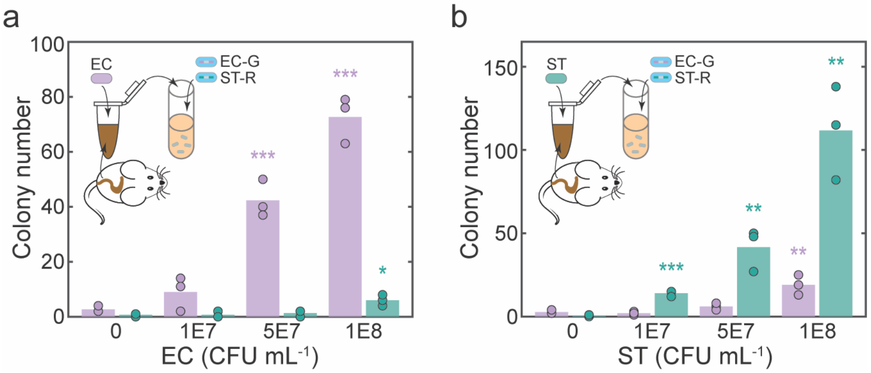

Supplementary Figure 10. Detection of spike-in *E. coli* or *S. typhimurium* in mouse ceca. Bar plot of colony numbers of EC-G and ST-R sensors cultured with 10 mg mouse ceca containing varying amounts of spike-in **(a)** *E. coli* or **(b)** *S. typhimurium* only. Mouse ceca were derived from germ-free mice orally gavaged with a synthetic microbial community. The sample (mouse ceca and spike-in target strain) was heat-treated and transferred to the mixed culture of EC-G and ST-R sensors. EC sensor can differentiate 5E7 and 1E8 *E. coli* cells per mL from no target cells (*p*-value = 5.73E-4 and 1.46E-4, respectively). ST sensor can differentiate 1E7, 5E7, and 1E8 *S. typhimurium* cells per mL from no target cells (*p*-value = 2.25E-4, 5.1E-3, and 2.4E-3, respectively). High density (10^8 cells per mL) of *E. coli* or *S. typhimurium* can lead to false positives of ST-R sensor (*p*-value = 0.0114) or EC-G sensor (*p*-value = 0.00983), respectively. Unpaired *t*-

test was performed to determine if there was a difference when compared to the negative control (no target cells in the sample), and *, **, and *** denote p-values < 0.05, 0.01, and 0.001, respectively. Bar represents the average of three technical replicates.

Supplementary Table 1. List of plasmids.

Plasmid	Description	Genotype
pAX01-comK[3]	Xylose-inducible *comK*	*lacA*(up), *erm*, P_{xylA}-*comK*, *xylR*, *lacA*(down)
pOSV00170	GFP reporter	*ycgO*(up), *cat*, $P_{hyperspank}$-*gfp*, *ycgO*(down)
pOSV00455	RFP reporter	*ycgO*(up), *cat*, $P_{hyperspank}$-*rfp*, *ycgO*(down)
pOSV00456	BFP reporter	*ycgO*(up), *cat*, $P_{hyperspank}$-*bfp*, *ycgO*(down)
pOSV00157	Kill switch plasmid without target sequence	*amyE*(up), *lacI*, $P_{hyperspank}$-*txpA-ratA*, *specR*, *amyE*(down)
pOSV00169	Kill switch plasmid with 500 bp EC homology	*amyE*(up), 0.5 kbp EC(up), *lacI*, $P_{hyperspank}$-*txpA-ratA*, 0.5 kbp EC(down), *specR*, *amyE*(down)
pOSV00205	Kill switch plasmid with 1000 bp EC homology	*amyE*(up), 1 kbp EC(up), *lacI*, $P_{hyperspank}$-*txpA-ratA*, 1 kbp EC(down), *specR*, *amyE*(down)
pOSV00206	Kill switch plasmid with 1500 bp EC homology	*amyE*(up), 1.5 kbp EC(up), *lacI*, $P_{hyperspank}$-*txpA-ratA*, 1.5 kbp EC(down), *specR*, *amyE*(down)
pOSV00207	Kill switch plasmid with 2000 bp EC homology	*amyE*(up), 2 kbp EC(up), *lacI*, $P_{hyperspank}$-*txpA-ratA*, 2 kbp EC(down), *specR*, *amyE*(down)
pOSV00208	Kill switch plasmid with 2500 bp EC homology	*amyE*(up), 2.5 kbp EC(up), *lacI*, $P_{hyperspank}$-*txpA-ratA*, 2.5 kbp EC(down), *specR*, *amyE*(down)
pOSV00292	Kill switch plasmid with 2500 bp ST homology	*amyE*(up), 2.5 kbp ST(up), *lacI*, $P_{hyperspank}$-*txpA-ratA*, 2.5 kbp ST(down), *specR*, *amyE*(down)
pOSV00459	Kill switch plasmid with 2500 bp SA homology	*amyE*(up), 2.5 kbp SA(up), *lacI*, $P_{hyperspank}$-*txpA-ratA*, 2.5 kbp SA(down), *specR*, *amyE*(down)
pOSV00475	Kill switch plasmid with 2500 bp CD homology	*amyE*(up), 2.5 kbp CD(up), *lacI*, $P_{hyperspank}$-*txpA-ratA*, 2.5 kbp CD(down), *specR*, *amyE*(down)

Supplementary Table 2. List of bacterial strains.

Strain	Description	Genotype
msOSV00487	EC sensor with 500 bp homology	*B. subtilis* PY79 *amyE*::0.5 kbp EC(up), *lacI*, $P_{hyperspank}$-*txpA-ratA*, 0.5 kbp EC(down), *specR*; *ycgO*::*cat*, $P_{hyperspank}$-*gfp*; *lacA*::*erm*, P_{xylA}-*comK*, *xylR*
mOSV00580	EC sensor with 1000 bp homology	*B. subtilis* PY79 *amyE*::1 kbp EC(up), *lacI*, $P_{hyperspank}$-*txpA-ratA*, 1 kbp EC(down), *specR*; *ycgO*::*cat*, $P_{hyperspank}$-*gfp*; *lacA*::*erm*, P_{xylA}-*comK*, *xylR*
mOSV00581	EC sensor with 1500 bp homology	*B. subtilis* PY79 *amyE*::1.5 kbp EC(up), *lacI*, $P_{hyperspank}$-*txpA-ratA*, 1.5 kbp EC(down), *specR*; *ycgO*::*cat*, $P_{hyperspank}$-*gfp*; *lacA*::*erm*, P_{xylA}-*comK*, *xylR*
mOSV00582	EC sensor with 2000 bp homology	*B. subtilis* PY79 *amyE*::2 kbp EC(up), *lacI*, $P_{hyperspank}$-*txpA-ratA*, 2 kbp EC(down), *specR*; *ycgO*::*cat*, $P_{hyperspank}$-*gfp*; *lacA*::*erm*, P_{xylA}-*comK*, *xylR*
msOSV00495	EC sensor (EC-G sensor) with 2500 bp homology	*B. subtilis* PY79 *amyE*::2.5 kbp EC(up), *lacI*, $P_{hyperspank}$-*txpA-ratA*, 2.5 kbp EC(down), *specR*; *ycgO*::*cat*, $P_{hyperspank}$-*gfp*; *lacA*::*erm*, P_{xylA}-*comK*, *xylR*

msOSV00605	ST sensor	*B. subtilis* PY79 *amyE*::2.5 kbp ST(up), *lacI*, $P_{hyperspank}$-*txpA-ratA*, 2.5 kbp ST(down), *specR*; *ycgO*::*cat*, $P_{hyperspank}$-*gfp*; *lacA*::*erm*, P_{xylA}-*comK*, *xylR*
msOSV00906	SA sensor	*B. subtilis* PY79 *amyE*::2.5 kbp SA(up), *lacI*, $P_{hyperspank}$-*txpA-ratA*, 2.5 kbp SA(down), *specR*; *ycgO*::*cat*, $P_{hyperspank}$-*gfp*; *lacA*::*erm*, P_{xylA}-*comK*, *xylR*
msOSV01005	CD sensor	*B. subtilis* PY79 *amyE*::2.5 kbp CD(up), *lacI*, $P_{hyperspank}$-*txpA-ratA*, 2.5 kbp CD(down), *specR*; *ycgO*::*cat*, $P_{hyperspank}$-*gfp*; *lacA*::*erm*, P_{xylA}-*comK*, *xylR*
msOSV01009	ST-R sensor	*B. subtilis* PY79 *amyE*::2.5 kbp ST(up), *lacI*, $P_{hyperspank}$-*txpA-ratA*, 2.5 kbp ST(down), *specR*; *ycgO*::*cat*, $P_{hyperspank}$-*rfp*; *lacA*::*erm*, P_{xylA}-*comK*, *xylR*
msOSV01008	SA-B sensor	*B. subtilis* PY79 *amyE*::2.5 kbp SA(up), *lacI*, $P_{hyperspank}$-*txpA-ratA*, 2.5 kbp SA(down), *specR*; *ycgO*::*cat*, $P_{hyperspank}$-*bfp*; *lacA*::*erm*, P_{xylA}-*comK*, *xylR*
usOSV00264	*Escherichia coli* MG1655	
usOSV00197	*Salmonella enterica* serovar Typhimurium LT2 ATCC 700720	
usOSV00113	*Staphylococcus aureus* DSM 2569	
usOSV00095	*Clostridium difficile* DSM 27147	
usOSV00165	*Staphylococcus epidermidis* ATCC 14990	
usOSV00046	*Clostridium hiranonis* DSM 13275	
usOSV00157	*Anaerostipes caccae* DSMZ 14662	
usOSV00011	*Bacteroides thetaiotaomicron* ATCC 29148	
usOSV00041	*Clostridium asparagiforme* DSM 15981	
usOSV00067	*Bifidobacterium longum* subs. *infantis* DSM 20088	

Supplementary Table 3. Sequences of genetic parts.

Part	Sequence
$P_{hyperspank}$-*txpA-ratA*	ctcgagggtaaatgtgagcactcacaattcattttgcaaaagttgttgactttatctacaaggtgtggcataa tgtgtgtaattgtgagcggataacaattaagcttacataaggaggaactactATGTCGACCTATG AATCTCTAATGGTCATGATCGGCTTTGCCAATTTAATAGGCGGGATTAT GACATGGGTAATATCTCTTTTAACATTATTATTCATGCTTAGAAAAAAAG

	ACACTCATCCTATTTACATTACTGTAAAGGAAAAGTGTCTACACGAGGA<br>CCCTCCTATTAAAGGGTAGTTTCTTTTTTAAAAGCTAGAGTGCTGCCAC<br>ACTCTGGCTTTTATATTTTAGCATTTCTCATGAAAGTAACACACATTAAC<br>AAGTGGTAATGTGGTAATGTGGTACCAACTATAAGCTTACGCCAGTAGT<br>TGCAATACTTTTGCTTGGCACCATTATAACATGAATATATATTGATTATA<br>TAATTATTTGTATCTTTTATTTGTTACTTTTTTTATCTATGAGTTCAAAATG<br>ACCTGATCATAGAAGCCTTAACCCTTTTTCTTTTATTAAAAACCCTCGGA<br>TTATGAAAGTGTTATGGTACAATATGGTTTAGTATAAATGAATATTGGCT<br>TTCAACATCTCAAGGGCGGTCTGGCTCACTCCCTCATGAAAGGGGGTG<br>ATGCACGTGTCAACATTTCAAGCATTAATGCTTATGCTTGCTTTCGGGT<br>CATTTATAATTGCCCTGTTGACTTATATAAAGAAGAAATAGACCCACCC<br>CTTGAGCTCGGCAAAGTAAAAGGGTAA
gfp(Sp)	ATGGTTTCTAAAGGTGAAGAATTGTTTACAGGTGTTGTTCCAATTTTGG<br>TTGAATTGGATGGTGATGTTAATGGTCATAAATTTTCTGTTTCTGGTGAA<br>GGTGAAGGTGATGCTACATACGGTAAATTGACATTGAAATTTATTTGTA<br>CAACTGGTAAATTGCCAGTTCCTTGGCCAACATTGGTTACAACATTTGC<br>TTATGGTTTGCAATGTTTTGCTCGTTATCCAGATCACATGAAACAACAT<br>GATTTCTTTAAATCTGCTATGCCAGAAGGTTATGTTCAAGAACGTACAA<br>TCTTTTTCAAGGATGATGGTAATTATAAGACACGTGCTGAGGTTAAGTT<br>TGAAGGTGATACATTGGTTAATCGTATCGAATTGAAGGGTATCGATTTT<br>AAAGAAGATGGTAATATCTTGGGTCATAAATTGGAATATAATTATAATTC<br>TCATAATGTTTATATCATGGCTGATAAACAAAAGAACGGTATTAAAGTTA<br>ATTTTAAAATTCGTCATAATATTGAAGATGGTTCTGTTCAATTGGCTGAT<br>CATTATCAACAAAATACACCAATTGGTGATGGTCCAGTTTTGTTGCCAG<br>ATAATCATTATTTGTCTACACAATCTAAATTGTCTAAAGATCCAAATGAA<br>AAACGTGATCACATGGTTTTGTTGGAATTTGTTACAGCTGCTGGTATTA<br>CACATGGTATGGATGAATTGTATAAATAA
mCherry	ATGGTTAGCAAAGGCGAAGAGGATAATATGGCGATCATCAAAGAATTTA<br>TGCGCTTTAAAGTTCATATGGAAGGCAGCGTTAATGGCCACGAATTTGA<br>AATTGAAGGCGAAGGTGAAGGCAGACCGTATGAAGGCACACAAACAG<br>CAAAACTGAAAGTTACAAAAGGCGGACCGCTGCCGTTTGCATGGGATA<br>TTCTGTCACCGCAATTTATGTATGGCAGCAAAGCATATGTTAAACATCC<br>GGCAGATATCCCGGATTATCTGAAACTGTCATTTCCGGAAGGCTTTAAA<br>TGGGAACGCGTCATGAATTTTGAAGATGGCGGAGTTGTTACAGTCACA<br>CAAGATTCATCACTGCAAGATGGCGAATTTATCTATAAAGTCAAACTGC<br>GTGGCACGAACTTTCCGTCAGATGGCCCTGTTATGCAGAAAAAAACAA<br>TGGGCTGGGAAGCATCAAGCGAAAGAATGTATCCGGAAGATGGTGCA<br>CTGAAAGGCGAAATTAAACAACGCCTGAAACTTAAAGACGGTGGACAT<br>TATGATGCGGAAGTCAAAACAACGTATAAAGCGAAAAAACCTGTTCAAC<br>TGCCTGGCGCATATAACGTTAACATTAAACTGGATATCACGAGCCATAA<br>CGAAGATTATACAATCGTCGAACAGTATGAAAGAGCAGAAGGACGCCA<br>TTCAACAGGCGGAATGGATGAACTGTATAAATACTAG
mTagBFP	ATGAGCGAACTGATCAAAGAAAACATGCATATGAAACTGTACATGGAAG<br>GCACAGTCGATAACCATCACTTTAAATGCACATCAGAAGGCGAAGGCA<br>AACCGTATGAAGGCACACAAACAATGAGAATCAAAGTTGTTGAAGGCG<br>GACCGCTGCCGTTTGCATTTGATATTCTGGCAACATCATTTCTGTATGG<br>CAGCAAAACGTTTATCAATCATACACAAGGCATCCCGGATTTTTTTAAA<br>CAATCATTTCCGGAAGGCTTTACATGGGAACGCGTTACAACATATGAAG<br>ATGGCGGAGTTCTGACAGCAACACAAGATACATCATTGCAAGATGGCT<br>GCCTGATCTATAATGTCAAAATTAGAGGCGTCAACTTTACAAGCAATGG

	CCCTGTTATGCAGAAAAAAACACTGGGCTGGGAAGCATTTACAGAAAC<br>ACTGTATCCGGCTGATGGCGGACTGGAAGGCAGAAACGATATGGCAC<br>TGAAACTGGTTGGCGGATCACATCTGATTGCAAACATCAAAACAACGTA<br>CCGCTCAAAAAAACCGGCAAAAAATCTGAAAATGCCTGGCGTCTATTAT<br>GTCGATTATAGACTGGAACGCATCAAAGAAGCGAACAACGAAACATAT<br>GTCGAACAACATGAAGTTGCAGTTGCGAGATATTGCGATCTGCCGTCA<br>AAACTGGGCCATAAACTGAATTACTAG
EC(up) 0.5 kbp	caattaccatcgaatgcaccattaacgggatgccttttcagcttcacgccgcaccaggcacgccgctctc<br>ggaattactccgcgaacaaggactgctaagtgtcaaacaagggtgctgcgtgggtgaatgtggtgcctgt<br>acggtgttggtcgacggcacagcaatagacagttgcttataccttgccgcctgggctgaaggaaaagag<br>atccgcacgctggaaggtgaagcgaaaggcggaaaactttctcatgttcagcaggcttatgcgaaatcc<br>ggcgcagtgcagtgcgggttttgtacgcctggcctgattatggctaccacggcaatgctggcgaaaccac<br>gcgagaagccattaaccattacggaaattcgtcgcggactggcgggaaatctttgtcgctgcacggggt<br>atcagatgattgtaaatacagttctggattgcgagaaaacgaagtaa
EC(down) 0.5 kbp	ttatgtgtttaacaactcatatttcttaatcttgcgatagagcgtagcaatgccgatgcccagttcatcagcaa<br>cttgcttcttgctgttatgacgtgaaagcgcctcgcggatcatttgcttttccatctcctccagcgccgtgccgc<br>ccgcatcatcgagtgacaggtgcgcctcactgacctctgttacatcactttgctccgttgtgccattattcagc<br>agatttggcggcaatagcgtgctgtcgataacttcacctgaaggaaccacgttaaccagatattccatca<br>aattgcttaactcgcgcaggtttccgggccaacgatgcttacgcaatatttcgacgacatcgggagcaatg<br>ccaggataaaccgatcccagacgacgggtatgcagatgtaaaaagtaatgcaccaatagttcaatatct<br>tcctgacgttcacgcagcggtggcagagttatcgggataacattaagtcggtagaagagatctt
EC(up) 1 kbp	atgacgcgaaactggagatccactccccgcgcggtgttcgtttcgtcccgattaatggctttcacaccggg<br>ccgggcaaagtgtctcttgagcatgacgaaatcctcgtcgcctttcattttccgccacagccgaaagaac<br>acgcgggcagcgcgcattttaaatatgccatgcgcgacgcaatggatatttcaacgattggctgcgccgc<br>acattgccgactggataacggcaatttcagcgaattacgcctggcatttggtgttgccgcgccaacgccg<br>attcgctgccaacatgccgaacagactgcacaaaatgcgccattaaacctgcaaacgctggaagctat<br>cagcgaatctgtcctgcaagatgtcgccccgcgttcttcatggcgggccagtaaagagtttcgtctgcatct<br>catccagacgatgaccaaaaaagtgattagcgaagccgtcgccgcggcgggggggaaaattgcaatg<br>aatcacagcgaaacaattaccatcgaatgcaccattaacgggatgccttttcagcttcacgccgcacca<br>ggcacgccgctctcggaattactccgcgaacaaggactgctaagtgtcaaacaagggtgctgcgtgggt<br>gaatgtggtgcctgtacggtgttggtcgacggcacagcaatagacagttgcttataccttgccgcctgggc<br>tgaaggaaaagagatccgcacgctggaaggtgaagcgaaaggcggaaaactttctcatgttcagcag<br>gcttatgcgaaatccggcgcagtgcagtgcgggttttgtacgcctggcctgattatggctaccacggcaat<br>gctggcgaaaccacgcgagaagccattaaccattacggaaattcgtcgcggactggcgggaaatcttt<br>gtcgctgcacggggtatcagatgattgtaaatacagttctggattgcgagaaaacgaagtaaaaggatat<br>ccggcctgaattcaggccggattcactg
EC(down) 1 kbp	aggttatgtgtttaacaactcatatttcttaatcttgcgatagagcgtagcaatgccgatgcccagttcatcag<br>caacttgcttcttgctgttatgacgtgaaagcgcctcgcggatcatttgcttttccatctcctccagcgccgtgc<br>cgcccgcatcatcgagtgacaggtgcgcctcactgacctctgttacatcactttgctccgttgtgccattattc<br>agcagatttggcggcaatagcgtgctgtcgataacttcacctgaaggaaccacgttaaccagatattcca<br>tcaaattgcttaactcgcgcaggtttccgggccaacgatgcttacgcaatatttcgacgacatcgggagca<br>atgccaggataaaccgatcccagacgacgggtatgcagatgtaaaaagtaatgcaccaatagttcaat<br>atcttcctgacgttcacgcagcggtggcagagttatcgggataacattaagtcggtagaagagatcttcgc<br>ggaatttaccttcggcaatgaactgggccaaattctgattagttgcagaaatgatgcgaatgtcgacttgta<br>ttgggctactggcaccaatcggcagaatttcacgtgcctcaatagcgcgcagtaatttagcctgcaacatt<br>aatggcatatcacctatttcatcgagaaacagcgtgcccgtattcgccgcctgaatcaaccctgttttaccg<br>ttggcagaagcgccagtaaatgcacctttaacataaccgaacagttcgctctccagaagctgctccgga<br>atcgcggcacagttgatagcaataaagggtttattccgtcttccgctcaacttatggattgcacgggcgac<br>gacttctttacccgtgccgctttcaccaaccaccataacgctggatgggctgggtgcaatacggctaatga<br>gtcgttttaattgccgcataacacggcactcgccaaccaattgttcaatatgcggttcatcaggtgcattt

EC(up) 1.5 kbp	cgtaacgcggtgaagatggctaccggtgttgcaatcaatacactgccgctgacgccaaaacggttatat<br>gaagagttccatctggcaggattgatttgaggataacatcatgtttgattttgcttcttaccatcgcgcagcaa<br>cccttgccgatgccatcaacctgctggctgacaacccgcaggccaaactgctcgccggtggcactgac<br>gtactgattcagctccaccatcacaatgaccgttatcgccatattgttgatattcataatctggcggagctgc<br>ggggaattacgctggcggaagatggctcgctacgtatcggctctgcaacgacatttacccagctaatag<br>aagatcctataactcaacgtcatctcccggcgttatgtgctgcggccacgtccattgctggaccgcagatc<br>cgtaacgtcgctacctacggtggaaatatttgcaacggtgccaccagcgcagattctgccacgccaacg<br>ctaatttatgacgcgaaactggagatccactccccgcgcggtgttcgtttcgtcccgattaatggctttcaca<br>ccgggccgggcaaagtgtctcttgagcatgacgaaatcctcgtcgcctttcattttccgccacagccgaaa<br>gaacacgcgggcagcgcgcattttaaatatgccatgcgcgacgcaatggatatttcaacgattggctgc<br>gccgcacattgccgactggataacggcaatttcagcgaattacgcctggcatttggtgttgccgcgccaa<br>cgccgattcgctgccaacatgccgaacagactgcacaaaatgcgccattaaacctgcaaacgctgga<br>agctatcagcgaatctgtcctgcaagatgtcgccccgcgttcttcatggcgggccagtaaagagtttcgtct<br>gcatctcatccagacgatgaccaaaaaagtgattagcgaagccgtcgccgcggcgggggggaaaattg<br>caatgaatcacagcgaaacaattaccatcgaatgcaccattaacgggatgccttttcagcttcacgccgc<br>accaggcacgccgctctcggaattactccgcgaacaaggactgctaagtgtcaaacaagggtgctgcg<br>tgggtgaatgtggtgcctgtacggtgttggtcgacggcacagcaatagacagttgcttataccttgccgcct<br>gggctgaaggaaaagagatccgcacgctggaaggtgaagcgaaaggcggaaaactttctcatgttca<br>gcaggcttatgcgaaatccggcgcagtgcagtgcgggttttgtacgcctggcctgattatggctaccacgg<br>caatgctggcgaaaccacgcgagaagccattaaccattacggaaattcgtcgcggactggcgggaaa<br>tctttgtcgctgcacggggtatcagatgattgtaaatacagttctggattgcgagaaaacgaagtaaaagg<br>atatccggcctgaattcaggccggattcactg
EC(down) 1.5 kbp	aggttatgtgtttaacaactcatatttcttaatcttgcgatagagcgtagcaatgccgatgcccagttcatcag<br>caacttgcttcttgctgttatgacgtgaaagcgcctcgcggatcatttgcttttccatctcctccagcgccgtgc<br>cgcccgcatcatcgagtgacaggtgcgcctcactgacctctgttacatcactttgctccgttgtgccattattc<br>agcagatttggcggcaatagcgtgctgtcgataacttcacctgaaggaaccacgttaaccagatattcca<br>tcaaattgcttaactcgcgcaggtttccgggccaacgatgcttacgcaatatttcgacgacatcgggagca<br>atgccaggataaaccgatcccagacgacgggtatgcagatgtaaaaagtaatgcaccaatagttcaat<br>atcttcctgacgttcacgcagcggtggcagagttatcgggataacattaagtcggtagaagagatcttcgc<br>ggaatttaccttcggcaatgaactgggccaaattctgattagttgcagaaatgatgcgaatgtcgacttgta<br>ttgggctactggcaccaatcggcagaatttcacgtgcctcaatagcgcgcagtaatttagcctgcaacatt<br>aatggcatatcacctatttcatcgagaaacagcgtgcccgtattcgccgcctgaatcaaccctgttttaccg<br>ttggcagaagcgccagtaaatgcacctttaacataaccgaacagttcgctctccagaagctgctccgga<br>atcgcggcacagttgatagcaataaagggtttattccgtcttccgctcaacttatggattgcacgggcgac<br>gacttctttacccgtgccgctttcaccaaccaccataacgctggatgggctgggtgcaatacggctaatga<br>gtcgttttaattgccgcataacacggcactcgccaaccaattgttcaatatgcggttcatcaggtgcatttgct<br>acagaaaaactggtatgcgattggtgaaacgccattaaaaataattgtcggccctgaatgttatgcaattg<br>accaatgattaattcacttttatcgtcccatgaaacaatatgctgcatatgtccatgggtaaaattactctcaa<br>atgttaatggtctgaaacggataggtttcccaataatattattttgcacaacaccaagtgtttttaaggcagtc<br>tgattaacaaactgaacccgattttcatcatctacaactaatacgccctgatccatattatcgatcatggtcg<br>caaatattttactgatgttatctcctggcccctgatcctccagaagtttcgaaacaaaaatggtggatatatg<br>gcgaacataatcagaaaattcgcgtaaattatcactgatatgctcttgttgctcgtgggtaacggcaatcaa<br>acttatcaccccaacacaacgatcctgtaaaatgacaggcgtacccagaaatgctttttc
EC(up) 2 kbp	ccctgataaaaggccatatcgtgctggttgaacgaccggaagagccgttaatgtcgttaaaagatttggc<br>gatggacgctttctaccaccctgaacgcggcgggcagctctctgctgaaagctccatcaaaaccaccac<br>taacccaccggcgtttggctgtacctttgttgatctgacggtcgatattgcgctgtgcaaagtcaccatcaac<br>cgcatcctcaacgttcatgattcagggcatattcttaatccactgctggcagaaggtcaggtacacggcgg<br>aatgggaatgggcattggctgggcgctatttgaagagatgatcatcgatgctaaaagcggcgtggtccgt<br>aaccccaatctgctggattacaaaatgccgaccatgccggatctgccacaactggaaagcgcgttcgtc<br>gaaatcaatgagccgcaatccgcatacggacataagtcactgggtgagccaccaataattcctgttgcc

	gctgctattcgtaacgcggtgaagatggctaccggtgttgcaatcaatacactgccgctgacgccaaaac<br>ggttatatgaagagttccatctggcaggattgatttgaggataacatcatgtttgattttgcttcttaccatcgc<br>gcagcaacccttgccgatgccatcaacctgctggctgacaacccgcaggccaaactgctcgccggtgg<br>cactgacgtactgattcagctccaccatcacaatgaccgttatcgccatattgttgatattcataatctggcg<br>gagctgcggggaattacgctggcggaagatggctcgctacgtatcggctctgcaacgacatttacccag<br>ctaatagaagatcctataactcaacgtcatctcccggcgttatgtgctgcggccacgtccattgctggacc<br>gcagatccgtaacgtcgctacctacggtggaaatatttgcaacggtgccaccagcgcagattctgccac<br>gccaacgctaatttatgacgcgaaactggagatccactccccgcgcggtgttcgtttcgtcccgattaatg<br>gctttcacaccgggccgggcaaagtgtctcttgagcatgacgaaatcctcgtcgcctttcattttccgccac<br>agccgaaagaacacgcgggcagcgcgcattttaaatatgccatgcgcgacgcaatggatatttcaacg<br>attggctgcgccgcacattgccgactggataacggcaatttcagcgaattacgcctggcatttggtgttgcc<br>gcgccaacgccgattcgctgccaacatgccgaacagactgcacaaaatgcgccattaaacctgcaaa<br>cgctggaagctatcagcgaatctgtcctgcaagatgtcgccccgcgttcttcatggcgggccagtaaaga<br>gtttcgtctgcatctcatccagacgatgaccaaaaaagtgattagcgaagccgtcgccgcggcgggggg<br>aaaattgcaatgaatcacagcgaaacaattaccatcgaatgcaccattaacgggatgccttttcagcttc<br>acgccgcaccaggcacgccgctctcggaattactccgcgaacaaggactgctaagtgtcaaacaagg<br>gtgctgcgtgggtgaatgtggtgcctgtacggtgttggtcgacggcacagcaatagacagttgcttatacct<br>tgccgcctgggctgaaggaaaagagatccgcacgctggaaggtgaagcgaaaggcggaaaactttct<br>catgttcagcaggcttatgcgaaatccggcgcagtgcagtgcgggttttgtacgcctggcctgattatggct<br>accacggcaatgctggcgaaaccacgcgagaagccattaaccattacggaaattcgtcgcggactgg<br>cgggaaatctttgtcgctgcacggggtatcagatgattgtaaatacagttctggattgcgagaaaacgaa<br>gtaaaaggatatccggcctgaattcaggccggattcactg
EC(down) 2 kbp	aggttatgtgtttaacaactcatatttcttaatcttgcgatagagcgtagcaatgccgatgcccagttcatcag<br>caacttgcttcttgctgttatgacgtgaaagcgcctcgcggatcatttgcttttccatctcctccagcgccgtgc<br>cgcccgcatcatcgagtgacaggtgcgcctcactgacctctgttacatcactttgctccgttgtgccattattc<br>agcagatttggcggcaatagcgtgctgtcgataacttcacctgaaggaaccacgttaaccagatattcca<br>tcaaattgcttaactcgcgcaggtttccgggccaacgatgcttacgcaatatttcgacgacatcgggagca<br>atgccaggataaaccgatcccagacgacgggtatgcagatgtaaaaagtaatgcaccaatagttcaat<br>atcttcctgacgttcacgcagcggtggcagagttatcgggataacattaagtcggtagaagagatcttcgc<br>ggaatttaccttcggcaatgaactgggccaaattctgattagttgcagaaatgatgcgaatgtcgacttgta<br>ttgggctactggcaccaatcggcagaatttcacgtgcctcaatagcgcgcagtaatttagcctgcaacatt<br>aatggcatatcacctatttcatcgagaaacagcgtgcccgtattcgccgcctgaatcaaccctgttttaccg<br>ttggcagaagcgccagtaaatgcacctttaacataaccgaacagttcgctctccagaagctgctccgga<br>atcgcggcacagttgatagcaataaagggtttattccgtcttccgctcaacttatggattgcacgggcgac<br>gacttctttacccgtgccgctttcaccaaccaccataacgctggatgggctgggtgcaatacggctaatga<br>gtcgttttaattgccgcataacacggcactcgccaaccaattgttcaatatgcggttcatcaggtgcatttgct<br>acagaaaaactggtatgcgattggtgaaacgccattaaaaataattgtcggccctgaatgttatgcaattg<br>accaatgattaattcacttttatcgtcccatgaaacaatatgctgcatatgtccatgggtaaaattactctcaa<br>atgttaatggtctgaaacggataggtttcccaataatattattttgcacaacaccaagtgtttttaaggcagtc<br>tgattaacaaactgaacccgattttcatcatctacaactaatacgccctgatccatattatcgatcatggtcg<br>caaatattttactgatgttatctcctggcccctgatcctccagaagtttcgaaacaaaaatggtggatatatg<br>gcgaacataatcagaaaattcgcgtaaattatcactgatatgctcttgttgctcgtgggtaacggcaatcaa<br>acttatcaccccaacacaacgatcctgtaaaatgacaggcgtacccagaaatgctttttcgcggcaatttt<br>ctttactatcgcaaccttcgcaaaggggatcgaagcgagactgtgtcacaactttttcagttttcgtttccagg<br>acgtggcggagcaggcgtgagttgccgctcaactggcgaccaagaaacttcccatacgcgcccgttcc<br>ggcaacgcgacacaagttttcatcaacgatctcaacctcaagctgcaaaacgctggcaagcattctggc<br>aaaacgctgaattgtcggttgaatttgcatcaatactgactgcgtagtagcaagctccatagctttaccttcc<br>agacttacttaaaagtcgatcattgaagacgttgatggttcacagatcatgatgatattaactcaggcgaa<br>attggctttgataaaaacataagattttatcattttctaatgaaattatggaagagatatcacatttctatatca<br>atatgagaattacggcggtgagtttatcaaactgaagagagatagcctgcccctttat

EC(up) 2.5 kbp	aggagatgctaatccgctcacgggcaaacgtatttacagcgcagggttgccggagtgtcttgaaaagg<br>ccggaaaatctttgaatgggaaaaacgccgtgcagaatgccagaaccagcaaggcaatttgcgccgc<br>ggcgttggcgtcgcctgttttagctacacctctaacacctggcctgtcggcgtagaaatagcaggcgcgc<br>gccttctgatgaatcaggatggaaccatcaacgtgcaaagcggcgcgacggaaatcggtcagggtgc<br>cgacaccgtcttctcgcaaatggtggcagaaaccgtgggggttccggtcagcgacgttcgcgttatttcaa<br>ctcaagataccgacgttacgccgttcgatcccggcgcatttgcctcacgccagagctatgttgccgcgcct<br>gcgctgcgcagtgcggcactattattaaaagagaaaatcatcgctcacgccgcagtcatgctacatcagt<br>cagcgatgaatctgaccctgataaaaggccatatcgtgctggttgaacgaccggaagagccgttaatgt<br>cgttaaaagatttggcgatggacgctttctaccaccctgaacgcggcgggcagctctctgctgaaagctc<br>catcaaaaccaccactaacccaccggcgtttggctgtacctttgttgatctgacggtcgatattgcgctgtg<br>caaagtcaccatcaaccgcatcctcaacgttcatgattcagggcatattcttaatccactgctggcagaag<br>gtcaggtacacggcggaatgggaatgggcattggctgggcgctatttgaagagatgatcatcgatgcta<br>aaagcggcgtggtccgtaaccccaatctgctggattacaaaatgccgaccatgccggatctgccacaa<br>ctggaaagcgcgttcgtcgaaatcaatgagccgcaatccgcatacggacataagtcactgggtgagcc<br>accaataattcctgttgccgctgctattcgtaacgcggtgaagatggctaccggtgttgcaatcaatacact<br>gccgctgacgccaaaacggttatatgaagagttccatctggcaggattgatttgaggataacatcatgtttg<br>attttgcttcttaccatcgcgcagcaacccttgccgatgccatcaacctgctggctgacaacccgcaggcc<br>aaactgctcgccggtggcactgacgtactgattcagctccaccatcacaatgaccgttatcgccatattgtt<br>gatattcataatctggcggagctgcggggaattacgctggcggaagatggctcgctacgtatcggctctg<br>caacgacatttacccagctaatagaagatcctataactcaacgtcatctcccggcgttatgtgctgcggcc<br>acgtccattgctggaccgcagatccgtaacgtcgctacctacggtggaaatatttgcaacggtgccacca<br>gcgcagattctgccacgccaacgctaatttatgacgcgaaactggagatccactccccgcgcggtgttcg<br>tttcgtcccgattaatggctttcacaccgggccgggcaaagtgtctcttgagcatgacgaaatcctcgtcgc<br>ctttcattttccgccacagccgaaagaacacgcgggcagcgcgcattttaaatatgccatgcgcgacgc<br>aatggatatttcaacgattggctgcgccgcacattgccgactggataacggcaatttcagcgaattacgcc<br>tggcatttggtgttgccgcgccaacgccgattcgctgccaacatgccgaacagactgcacaaaatgcgc<br>cattaaacctgcaaacgctggaagctatcagcgaatctgtcctgcaagatgtcgccccgcgttcttcatgg<br>cgggccagtaaagagtttcgtctgcatctcatccagacgatgaccaaaaaagtgattagcgaagccgtc<br>gccgcggcgggggggaaaattgcaatgaatcacagcgaaacaattaccatcgaatgcaccattaacgg<br>gatgccttttcagcttcacgccgcaccaggcacgccgctctcggaattactccgcgaacaaggactgcta<br>agtgtcaaacaagggtgctgcgtgggtgaatgtggtgcctgtacggtgttggtcgacggcacagcaata<br>gacagttgcttataccttgccgcctgggctgaaggaaaagagatccgcacgctggaaggtgaagcgaa<br>aggcggaaaactttctcatgttcagcaggcttatgcgaaatccggcgcagtgcagtgcgggttttgtacgc<br>ctggcctgattatggctaccacggcaatgctggcgaaaccacgcgagaagccattaaccattacggaa<br>attcgtcgcggactggcgggaaatctttgtcgctgcacggggtatcagatgattgtaaatacagttctggatt<br>gcgagaaaacgaagtaaaaggatatccggcctgaattcaggccggattcactg
EC(down) 2.5 kbp	aggttatgtgtttaacaactcatatttcttaatcttgcgatagagcgtagcaatgccgatgcccagttcatcag<br>caacttgcttcttgctgttatgacgtgaaagcgcctcgcggatcatttgcttttccatctcctccagcgccgtgc<br>cgcccgcatcatcgagtgacaggtgcgcctcactgacctctgttacatcactttgctccgttgtgccattattc<br>agcagatttggcggcaatagcgtgctgtcgataacttcacctgaaggaaccacgttaaccagatattcca<br>tcaaattgcttaactcgcgcaggtttccgggccaacgatgcttacgcaatatttcgacgacatcgggagca<br>atgccaggataaaccgatcccagacgacgggtatgcagatgtaaaaagtaatgcaccaatagttcaat<br>atcttcctgacgttcacgcagcggtggcagagttatcgggataacattaagtcggtagaagagatcttcgc<br>ggaatttaccttcggcaatgaactgggccaaattctgattagttgcagaaatgatgcgaatgtcgacttgta<br>ttgggctactggcaccaatcggcagaatttcacgtgcctcaatagcgcgcagtaatttagcctgcaacatt<br>aatggcatatcacctatttcatcgagaaacagcgtgcccgtattcgccgcctgaatcaaccctgttttaccg<br>ttggcagaagcgccagtaaatgcacctttaacataaccgaacagttcgctctccagaagctgctccgga<br>atcgcggcacagttgatagcaataaagggtttattccgtcttccgctcaacttatggattgcacgggcgac<br>gacttctttacccgtgccgctttcaccaaccaccataacgctggatgggctgggtgcaatacggctaatga<br>gtcgttttaattgccgcataacacggcactcgccaaccaattgttcaatatgcggttcatcaggtgcatttgct

	acagaaaaactggtatgcgattggtgaaacgccattaaaaataattgtcggccctgaatgttatgcaattg<br>accaatgattaattcacttttatcgtcccatgaaacaatatgctgcatatgtccatgggtaaaattactctcaa<br>atgttaatggtctgaaacggataggtttcccaataatattattttgcacaacaccaagtgtttttaaggcagtc<br>tgattaacaaactgaacccgattttcatcatctacaactaatacgccctgatccatattatcgatcatggtcg<br>caaatattttactgatgttatctcctggcccctgatcctccagaagtttcgaaacaaaaatggtggatatatg<br>gcgaacataatcagaaaattcgcgtaaattatcactgatatgctcttgttgctcgtgggtaacggcaatcaa<br>acttatcaccccaacacaacgatcctgtaaaatgacaggcgtacccagaaatgctttttcgcggcaatttt<br>ctttactatcgcaaccttcgcaaaggggatcgaagcgagactgtgtcacaactttttcagttttcgtttccagg<br>acgtggcggagcaggcgtgagttgccgctcaactggcgaccaagaaacttcccatacgcgcccgttcc<br>ggcaacgcgacacaagttttcatcaacgatctcaacctcaagctgcaaaacgctggcaagcattctggc<br>aaaacgctgaattgtcggttgaatttgcatcaatactgactgcgtagtagcaagctccatagctttaccttcc<br>agacttacttaaaagtcgatcattgaagacgttgatggttcacagatcatgatgatattaactcaggcgaa<br>attggctttgataaaaacataagatttttatcattttctaatgaaattatggaagagatatcacatttctatatca<br>atatgagaattacggcggtgagtttatcaaactgaagagagatagcctgcccctttatcttatttctgatactt<br>agcagcaaataaataacgcgataaaaaaagccaaacgttttcgtattttacaaacaaccagaagctgg<br>catcaatttgtgatcaaccccacacattatccgtcaaattagtcttttgcagccgcgcggataattctggcac<br>acttattgttagtcccaggtatagctgtgaaaacaccaatcactttggcaagtcacagtgaaataaaccac<br>tttgcctgtcattccactaccgggactttatgatgaaaactgttaatgagctgattaaggatatcaattcgctg<br>acctctcaccttcacgagaaagatttttgttaacgtgggaacagacgccagatgaactgaaacaagtac<br>tggacgttgccgcagcattaaaagcactgcgtgctgaaaacatctcaaccaaagtctttaatagtggatta<br>ggtatttccgtattccgcgacaactccacccgtacccgcttctcttatgcttccgcg
ST(up) 2.5 kbp	acgtagcagcaggggtatcaacgtttgcatttcaaggtgccgggcttcccgtcctacgctggtaccctgct<br>cttgcgttaatttttggtggcacatatcaagcgcctcaacagccttcgccgccgctttgtcaacaaggtgcgt<br>aagattgctgcgggttaacggatctaacgtacagccaaagttatgttcaatgcagctggcaatatagggc<br>atcacctcctgcataacaagattcgtcgataatttacttaattcaccgccagtgttatttttgataatatctaac<br>agctgcttttccaggttttccagcttcgcttccgctttctttgtttctggcagccatggcccaaaagctgacttttct<br>ttcaggccatcttttatgatttgcgcggtatactctgcccccaccttcatcagtagcgtcttcgcctcaggaga<br>atcactggtggcgttgagcgctgaacgaaagagcccggcaaactccattatcgctttcttaccggcgaca<br>ttatttgaattggtaaaaacttcttttaacgcctcagcgtctttcccgcatttaaacaatgcatccagactcgcc<br>tgtttgatcagcgcgggaaaatcttccagttgcgggcctttaatttcccctgacagcgtcgctgtggcactttc<br>tctgactgcggaaagattcgccgcaagattcgtggcctgcgttttgatctcggtctgcatacctggcattatg<br>acggggggctgagtccttacacttgtaaccattattaatatcctcttctgttatccttgcaggaagcttttggcg<br>gtttccaggctgctacttatcgtactgctcagcacttttaccaggttgtcgtacaatgaattggcattgctatatt<br>tttgcgtcagcgtctgtaatgtggttttcatattttcttcctgcgctttaaaacccgactgccaggcttgatatttg<br>gcgttatccatttcgagttttgagtcttttcccggcgcgcctaaaccatcaatatcctgaaccatttttttgtaatg<br>gcgtcagatcaacggtgacgacataaccggatccataagatttcaggcagctattcggtaaattcaattc<br>actgagccactgtctcgcttccgcttcagtggctactttaacgccgctgcctgactgcgctggaaataaaac<br>ggtattactgtttatttgattatatttattgactaaactgtttaaatcatttttgagtgaggtaacatctagcttaacg<br>gtattaccgtccttacctggtaataaccagcctcccattttggaaagaatatcactgaaggcctgataaaa<br>atcggtatagactgcgacaacgttttcataaacgcccagatagctgtcacctatcgccgatatattttggga<br>aaccatatcccaaatctcagcatcagaaatggttgttctcggctgcgccataggcgaagcgctaaataag<br>gccgacgtcggcgcagaaaacgcgctccgcaggttctcattttgttctgcggataatgacacgccggact<br>tcgccagcgcattcaggctgctggtcaactgctggcgcgccagcgtgcgctcgtcattattctcttcagaga<br>tcggtggcgttgactgcagcgtctgctgtgcctggtggattttagtagccgcctgcgataatgaaatgatatc<br>tgtaccgcgatgttctgtggtagacggtaccacggcagtctcgacgtgctcgctcgccgagggagtctgc<br>ggccgttcggcaacgatccccggatgaggagaagcggaataattttgaatattaagcataatatcccca<br>gttcgccatcaggagcgcgattaaatcacacccatgatggcgtatagatgacctttcagattaagcgcga<br>atattgcctgcgatagcagcgagtgcggatgctttcgactggttaatgctctccattgttttcagcatttcctga<br>atcaggctggtcgatttacgtgaactttcacgggcttcgtccgatgcggtgctggcaacccggttattcacct<br>ggctaatttgctgctcggaacgttcctgagtagcggcgtactgcccggacgcccctgcaataccaccgac

	cgtgaccgagttcttcataatcagatcgcccgtcatctgcatcttgcgcgcatcgattcgggtcatatccatg<br>gtattctgctcaagacgaatatcggattcgacagactcaagacgtttcgacagaatagcctgatgttcagg<br>ggagatttgtttattactgtctttaatacccagactttccgtggcgctggttccggcattagatttaagcgtcgc<br>atcattaagatttttcgtcgcatcggtaccggttttcttcatatttaacgatttcagagaatcgacgccttcagc<br>accgagtttgacgctattctgcccgttcagcacgtttttaatactgtggctttcagtggtcagtttatcgatcttcg<br>cggcattatgtttaag
ST(down) 2.5 kbp	cgcgcctctttcattctgcagccccttatattccagtttggcgcccacgccagtgatccccaactgaagcgc<br>gctctgggaaatactaccggacaacgcattcatcccttcgcgcatcatggagcttgccgtcgttttagctgc<br>atcaaaactgactaatgacaacttaccagacagtttgctatcagcctggttcaacgtcagcattaacgtatt<br>cgcggcagccaacagcgcaacggcactggaagacattccgctaatatcaaaaaactttccgacttctg<br>cctgctgctcgcgtaactgggtttgcacaacctcattcgctttagtcgtgacattatttgccagagcattcaaa<br>tcctgattcatgtcggtattttgaatactggcttttaaaaaggacgtgatcgttccgggggtttgcgttaatacc<br>cctggcgcaggcgcgctcagtgtaggactcaaccccaggtcactgactttactgctgctaataccaatact<br>attcagaatatctttagcgctaacggattgcgaagctgtctgtgaactattctcaacagaatgattatttaaat<br>aagcggcgggatttattcccacattactaattaacatatttttctccctttattttggcagtttttatgcgcgactct<br>ggcgcagaataaaacgcgaagcatccgcattttgctgtaccgcagaagacatggctttttgcagttccgc<br>cgttaccttctggttttcaccaaatatttctacggattgtttaagccactgctgaatctgatccatggcaaaacg<br>ggcgagcataaaatcagcaagcgcctcgctggcatttttaataaatacgccctcggcaacaccaccgg<br>ctgactgggctgcggtattcgtgacttccatgcccaacgccactttatttagggtattacctaccagctcttta<br>cttaaggcattcgtttgcaggcccatcttgctacccacattacccagaccgctagtaatacgttgcatcccct<br>gggtaaagagtttgctgccgttttgcgccaactgtttcagcacgttaggcaccaacttcttaatcgtttcgccc<br>atcattttgctcagcgcgttacccagtttcgccgccgcgcctttcccgacaactgcgaccaccacaatgac<br>cgccaccatggcaatagcggcgacaatcgcaccaacaatgctgccggccatctctgccgttttcttatcg<br>acgcctaatccttccagcgctttggtaatcgccttgccaatcagctccattaacggcttcagcacatgctcc<br>ataatcgggtttagcgcctgctgaataaacgacactcccgtcgccgccttcacaatttcatcggccaccatt<br>accgcaagtcccaccgcagccagcgccagactcgccccaccggtaaaaacagcggccacaacgct<br>gacaatggttagcagcgcgccgaggactttcccgatacatcccataatgcggttcgtttcctcggctttgcg<br>cgtctcttcctggaattcagccgatttcttttccatctccgcctgacgcccttcctgcaaggcgttgaaaagcg<br>caagatcgttttgcaggctttcttccgtatttttgcccacaatctcaataaacatggccatgagcatagtgag<br>gcgggcgacatttgacagattatcctgctcaccctgggaaacctgattctgagaggcggcattagccgttc<br>cctggaatttggtcagaatgttatccgctttctcggctttcgctttggcgtctgtgcctgctttaaccgtcgcatc<br>cgtggccttatctaaggcctctttcgcctctgtcgcttcttttccggcctgttctaccgcggcttcagcttgtgcat<br>agccggggtcagccgggtccagcgattgcaatttattttgcgcctgcgtcagtttttttggtcgcagcgtcata<br>aacactcttggcggtatccgtctttttgatactggcttcatagagatccgtcgcctcctgagcctctcccagag<br>ccgtctggaattctttcgatacctgaatccccatctctttttgtgactcaatcatcgcctgccataccgccaga<br>cgagactccagttgagacagcgaaacatcgcccagtagggtcattaacttgccaagcagtaatgtcaat<br>tgcccttcgctggagagtttttcccgggcggcgtccgtaggcggctttagacccaccgtattaatagcgctc<br>tcgccggactttgttccggctttaaggtcgcccgctttcgttgccaccacatctttaaaagctttatccgccgct<br>tttaaaaagtccgtgttcttacgaacgccttcaaaagccgcctcagcgaggcgcggattttgggtatatccg<br>ctacggctaatgctacttgcgtcatttaccataattattccttttcttgttcactgtgctgctctgtctccgccgttttt<br>agcgcctccagatagaccaacgcttttg
SA(up) 2.5 kbp	ctatcgtgtcatgtaatcttgcatccgatcttgcaacgctgtaaatgtttcgaagccatcctcttctaagaagtg<br>ccctccatcttccacgattcgcaagttcccctctaatgcattcattaaacgctgggtttctttatatgaaacgta<br>tttgtcatttttagaactcaatccgtaaaaattgtcaactttctttttaatattatcgtaatcaatggttacattactt<br>aaatcaatatctaaatctatattttctgcatcttctttaaagcccgctatactaaaaaagccttcaatcggctg<br>atcaatcatttcaatatattttaaagctgtgattgaacctaaaccatgtgttacaaaatatgtatccttttttgcgt<br>acattaatttgtttcgtcatagcttcaatccactgatccactgtcttcgcttcaggggattcaaaattaaataat<br>gttacgtcatatccttctaaagttaagttatgctccaaccactgataccaatgatttctactatttccatgcatag<br>aatgtacaataattacatctgtcatctcattctctcctttcaacttactacttcttttctatttttaaaaaaatgactg<br>attacctataattgtaaaataaaaacaccttaattagaaatgttatatcgcaaagtgacatttctaattaaagt

	gtattgtcatcatttcaatatcattcaaaaacagctaaacctttgtctctgcttcaatttcacaaaaataattccc<br>gctgaaagtatctatatttacacattacttccaccattatataacttaaaaatgactatatttcatcaaacattat<br>ctaaaggcgtcgcacctacaccaacaccatccaacaattaacttacaactctgcgattacttcttcagcag<br>caactttaccttgtgtaatacaatcaggtagtccaaccgcttcaaaagatgcaccagttactctaagtcgtg<br>gatatgtttgtttaatatgtgcttgaatctgtctaatttgttgaatatgaccgacatggtactgtggcatacttttcg<br>gcaaacgattgacaattgtaaattcaggatcacctttaaatgtcatcatttgacttaaatctctacgtacaatc<br>gatactaattcattatctgtatgatcatcaaccacagtatcacctggtttacctacatacgcacgaatcaaaa<br>ccttaccttctggtgtagtaaatggccatttttcgatgtccaagtacatgcggtaatgtctgtatcactcgttctc<br>gcaatcacgaagccagtaccatcataagtattttcaatgtcttttcatcaaatgccaatacaacagttgca<br>acagtcgtactatccatcgttttaaagtaatcaaatgctggatcttgtccgaaccaattcaaaaagacttgat<br>gcggtgttgtcactaataccccatcgaatacatcttcttgttgattactgtaaacaattttatattgcttttgagat<br>gtaataatatcatccactgacgtattgtagcgtattgtcacacctttatttttcacatcttgttctaatgcttcaata<br>aatgagcttaaaccatgcttaaattgtttgaattgtccttttggtgcgccaggatataattgtctttgtttcagac<br>gcttatttttctcatccttcatacctttatcagacttccgaatgcctcttctttttctttaaaattaggaaacgtactc<br>atcaaacttaatttatcaatatcggtaccataaataccacccattaaaggctcaattaagttctcaagtacct<br>cattacctaatcttgctctgaaaaatgcaccaacagaaatgtcaccatcttgcatttgtataggctttttgatta<br>aatctaatcctgctcttaatttaccaagtggcgatattaattttgtagtaacaaatggtttaatatctgttggaat<br>acccataattgaaccacctggaatcggatataatttatttttcgcaaaaatatatgattgtccagtcgtatttgt<br>aacaatatcttgttctaatccaatatctttcgctaattctgtcataatcgtttttctacctaaataagattcaggcc<br>ctagttcaatcatataaccatctttacgatacgattgaatctttcccccggacgattcgatgcttcaaagatg<br>gttacatcaatattaggatcttgctgttttaa
SA(down) 2.5 kbp	gacttgatttcatcaacaattgcaccgataaataatggatgtgtattcggcatttttggacgataataattcgc<br>accaatatcatcgcaaacaactttacattcataatcattgtcataaagcacctctaaatgctcacatacaaa<br>acctactggcgtatatataaagtttttatactgatgtttttcatataaatcacgtgttaaatcttgtacatctggcc<br>ctaaccaaggtgtacctgtattaccttcagattgccaaccaatcgcgatatgttcaatattagattgttctttaa<br>ttaaaagcgcagtatgttctagttcttgtggatatggatcattattcttttcgattaaaccttttggcaaactatgt<br>gccgaaacaactaataccgtgtctttatgttcctcttccggtatttgagctaatgtttcgttgactttattcgtcca<br>atattcaataaatttaggttgttcataataatgtttcacatgtgtaagttgaataccatattttgcagcttcttcatc<br>agcacgtttgtcatatgatcctactgaaaatgaagaataatgtggtgctagtactaccgtgattgcttcagta<br>ataccatcattgtgcatttgttcaaccgcatcttcgataaatggtgaaatgtgttttaatcctaagtatagtttaa<br>attcaacatctgcatatgctttatttaatgctgaaactagtgcatcagcttggtcatctgttgtacctgctaatgg<br>tgataaaccacctataaattcatatctatctttcaaatcttgaagttcttcttcagatggacgtttaccatgtcta<br>atatctgtataatatggctctatgtcactttctttataaggtgtgccataagccataactaataaccccattttttt<br>agtcattgataataccttcctttaaatgaattatctttcatgtgcttcaatgtaatactatgattatctttgtgtatat<br>gtgtgtacgaattcgcttactttacgtaacgtctctggttgcacttctgggaaaacaccgtgtcctaaattaaa<br>gatgtgtttaccgttctccataccttgatctaatattggtttcaatctctcttcaatgacattccatggtgctaata<br>aaattgatggatctaaattcccttgtaatgttttagtaacgcctaattgttgagcctgattaatagacgttctcca<br>atctaggcctaatacatcaatcggtaaatcattccattcattgattaaatgactggcacctacaccgaataa<br>aattaccggcacatcatgtttttctttaacctcactgattaatcgaatcatatgtggtttaatgtaacgtctgtaat<br>cctcgacatttaatgcacctacccatgaatcgaaaatttgaatcaattcggcacctgcttcgacttgagctgt<br>tacatatttaacagatacatcaactaaatgattcattaaagcaaaccatgttgcttcatctctatacatcatcg<br>cttttgtaaaattgtaatttttcgatggtccgccttcaatcatatatgacgctaatgtaaatggtgccccagtaa<br>atcctattagcggcacatttaacttttcttctgttaaaagtttaattgtatctaatacatatggtacatctcgttcgg<br>ggtctatttgagaaagtttctcaacatcttgaattgttttgataggattatgaatcactggaccaatacccgatt<br>taatttctacatcgacaccaattggctttaatggtgtcataatatctttgtataaaattgctgcatctgtatgata<br>attatcaactggtaaatgtgttacataagcgcacaactccggctgatgtgtaatatcgaatagtgaatattttt<br>ctttcaattttcgatattctggttgcgaacggccagcttgtcgcataaaccaaacaggtgtatgtgatgtttctt<br>cacctttgatcattttaaaattgtattgtttttattatgcaccataaaggcctcctaaattaaaatcattcttatcta<br>tattatcatatcgctcattcgttcgtattttcaataaataaatgtcataaaactgacatttaatcatagaactattt<br>attgtaaatttaaattctaaagtccattattttgtatcattacttctaaatatctcgcaagattcattatagtaatttt

	aatcaattattaatagtggtaatgactagtttatcatcgtataataaataaaaacataaggggacctttcat<br>atgaagaaactatatacatcttatggcacttatggatttttacatcaaataaaaatcaataacccgacccat<br>caactattccaattttcagcatcagatacttcagttatttttgaagaaactgatggtgagactgttttaaaatca<br>ccttcaatatatgaagttattaaagaaattggtgaattcagtgaacatcatttctattgtgcaatcttcattccttc<br>aacagaagatcatgcatatcaacttgaaaagaaactgattagtgtagacgataatttcagaaactttggtg<br>gctttaaaagctatcgtttgttaagacctgctaaaggtacaacatataaaatttatttcggatttgctgatcga<br>catgcatacgaagactttaagcaatctgatgcctttaatgaccatttttcaaaagacgcattaagtcattactt<br>tggttcaagcggacaacattcaagttattttgaaagatatctatacccaataaaagaatag
CD(up) 2.5 kbp	atgaagcaatatatagtcattgggtgtgggagatttggaagttcagttgcgtctactatgcatcttttaggaca<br>tcaagtaatggcaatagacaaaaatgaagattcagttcaaagtatatctgacaaggtaacccattcactt<br>atagtggatgttactgatgagcaagcgttaaggtcattaggtttaggtaactttgatgtagcagtagttgcaa<br>taggttctgatataagggcatctataatggcgactcttatagccaaagaaatgggtgtagagttgataatat<br>gtaaggcaaaggatgaattacaagctaaagtgctttataaaattggcgcagatagagttgtatttccagaa<br>agagatatgggagtaagagttgcacacaatttagtttcggataatatattagaccatattgaacttgaccca<br>gagtattcaattgttgaaatcgtaactccaaatagttgggttggcaagacacttatagagcttgaattaaga<br>gctagatatgagataactgtacttgctataaaaacaggtaaaaatataaatgttacaccttctccagatga<br>ggaacttacagccggaagtatcctagttataatcggtcaaaatactagtataacagcgataacatctgga<br>aataaggggataattagaagaagataatttactatttaatatatatttaattgcaatgaaagtaaagagtatc<br>atataattatgaatagttatatgatactatttttatttaatcgaaggtagtatttatttgtaagattagataaagag<br>aagttaaattaaaagtaaggaggctgtgctaatataaaaatttataagttattagcatttagataaaatgatt<br>acaaatataaacagtaaggataatgaaaagttaaagtatacaagagcactattaaaatcaaagaatag<br>gaataaagagtcaaagttcataatagaaggatacagaatagtaatgcttgcacttgaatgtatggcaaac<br>cttgattatgtatttatcaatgaagaatttgaaaataagaaagaacatgtaaaactattagaagatttggata<br>aaaaaaacacaaagatatacaagactactaataaaaactttaaagaattagtggatacagaaaatact<br>caaggaataataggtgtagtttcatttaagaaaaaaaaaattaagtgaaagtataaataaaaaagataaa<br>tttgtattgattttagatagaatacaagacccaggaaatatggggactataataaggactgctgattctgctg<br>gagtagatgccataatagcactaaagggatgtgtcgatatatacaacccaaaagtaattaggtctactat<br>gggttctatttttgatatgaatataattgatgcttcacaagatgaaactgtggacatgcttaaatcattggatttt<br>aatatagtttcaagttacttaaatacagaaaatttttatgacaaaatagattatggttcaaaagtagcattggt<br>gataggaaacgaagcaaatggaataaatgaagaacttgtatcaaagtctgatattttggttaagatacct<br>atatatggtaaagccgagtcgttaaatgctgcgataagctctgctatactgatgtatgaaataaaaaaata<br>cttaatttaatgtattgtaataaatataatgtcatgttataatcttgaattaaatagtagagtatcaaaaatagtc<br>aatatatattaaaaaaataattaaattaattatatattagatgtatatcatataaaaaatgattgagttgattat<br>gtattagatatatattatataaaaaataattgagttaattatgcattagatgtatattatataaaaaataattga<br>gttaattatgcattagatatatattatataaaaaataattaaattaattatgtattggatatatattaaaaatagt<br>cacataatttgaatggaaatgatataatactaaaataaacaatataatattgtaaatgcaatgaaagagg<br>aaagtattttgattaaacttagtaaagagataaacacctaggctgggagtgttttctaagaggtcatgaga<br>agttccctctggagtaacagagctgaaattttacagtaggctttgacgtcaaaaacgcgttaagtttgttag<br>aggtggtttgatgatttttaaattgttaaactactagggtggtaccgcgaaactata
CD(down) 2.5 kbp	tagacagggattgaggggctttttttatacaaaaaaacgaaagggtgatatgtgtgcaagaaaaattactt<br>gctttacgtgaagcagctttggctgaaataaaagaagcacaaagcatagaaagtgtagaaagtttaaga<br>gttaagtacttaggaaaaaaaggtgagataactgccatacttaaagaaatgggtaaattatctgctgaag<br>aaagaccagtagttggtaaggttgccaatgaggtaagagaaaacattgaacttagcataaattctaaaa<br>aagaagaaataaatgctattgaaaaagaaagaaaattaaaagaggaagtgatagatgttactcaacc<br>aggaaaagttttaaaggttgggaagaagcatccaataactcaaattatagatgaagtaacagatatattt<br>atcggaatgggattctctatagcagaagggccagaagttgagactgttgaaaacaactttgacgcattaa<br>acgctcctaaagaccatccatcaagagatatgagcgatacattctatatcaatgatggggtattacttaga<br>actcaaacatctccagttcaagtaagaactatgagaagtcaagagttaccaataaaagtaattgcacca<br>ggtagatgttttaggtcagactcgccagatgctacacactcaccaatgttccatcagatagaagggcttgtt<br>gttggaaaagatgttactatggcagaatttaaaggaactatggatatcttcgttgaaaaattgtttggttctga

	tatcaaaactaagtttagacctcacaacttcccatttacagaaccaagtgcagaggttgatgttacttgtttc<br>aaatgtggtggtaaaggttgcccaatgtgtaaatatgaaggttggatagaaatattaggttcaggtatggtt<br>catccaaatgtgcttagaaattgtggaatagacccagaagtttacagtggatttgcatttggagttggggttg<br>aaagacttgcaatgcttaaatacgaaatagatgatattagattattattcgaaaatgatatgagattcttaaa<br>tcaattttaattaggagggtatgtagatgttagtatctttaaaatggcttagagactatgttgatatagacatgg<br>atgtaaaagagttcgctgataaaatgacaatgacaggaactaaagttgaaacaatagattattatggtga<br>agaaatagaaaatatattggttggaaagattttagaaataaaacaacatccaaatgctgataagttggttg<br>taactaaagtagatattggagataaagttgttcaaatagttacaggagctacaaatatatcagaaggaga<br>ttatattccagtagctgtaaatggttctaagttacctggaggagttgaaatcaaacagactgatttcagaggt<br>gaattatcagatggtatgatgtgttcagcagctgaactaggtatagatgaacattacattgaggagtataa<br>aagaggtggtatatatattttagaccacgaagattcttatgaattaggaaaagatataaaagatgttttagg<br>attaaaagatgctttaatagattttgaattaacttcaaacagacctgattgtaaatgcatgatgggtatagcta<br>gagaagcagctgcaactataggaacaaaagtaaaatatcctgaaatcgaagtaaaagaaagtgacg<br>aagagatagatttcaaagttgagatagataatccagatttatgtagaagatatgttgctagaatggttacag<br>atgtaaaaatagaaccttctccatattggatgcaaagaagacttacagaagcaggagtaagacctataa<br>gtaacatagtcgatataacaaacttcgtaatgttagagcttggtcaaccacttcatgcttttgatataaatcaa<br>gtagagactggaagaatagtagtaagaaatgctaaagatggagagaaacttgtaacattagatgatgtt<br>gagagaacattagataaagatatgctagttataacaaatggagaaaaatcacttggtttagctggtgtaat<br>gggtggtgctaactcagaaataacttctaatacgaagactgtactttttgaaagtgccaatttcaaaccaga<br>aaacataagaatgacagctaaaaaagttggtattaggtcagaagcatcttcaagaaatgaaaaagactt<br>agaccctaatcttgcagagatagcagcaaatagagctgcacaacttgttgaaatgttaggagcaggaa<br>aagttttaaaaggtgttgtagatgtatatccaaataaaccagaacctaaaaaattggtagtaaatcctcaa<br>agaattaaccacctattaggtgtagatgtaccaatggagcagtttgtaggaattttagaatcattagagttta<br>aatgtaatttggtagctaatgataaattagaaatagatgtaccaagctttagaacagatatggaacaaga<br>agctgatgtatgggaagaaatagctagaatttatggatttgagaata

Supplementary Table 4. Sequences of primers.

Part	Sequence
EC_FW	GCGGACTGGCGGGAAATC
EC_RV	AATGATCCGCGAGGCGCTTTC
lacI_txpA_FW	ATCCTAACTCACATTAATTGCGTTGC
lacI_txpA_RV	CTTGAGGCATCAAATAAAACGAAAG

References

1. Overkamp, W. *et al.* Benchmarking various green fluorescent protein variants in Bacillus subtilis, Streptococcus pneumoniae, and Lactococcus lactis for live cell imaging. *Appl. Environ. Microbiol.* **79**, 6481–6490 (2013).

2. Tack, D. S. *et al.* The genotype-phenotype landscape of an allosteric protein. *Mol. Syst. Biol.* **17**, (2021).

3. Zhang, X.-Z. & Zhang, Y.-H. P. Simple, fast and high-efficiency transformation system for directed evolution of cellulase in Bacillus subtilis. *Microb. Biotechnol.* **4**, 98–105 (2011).
