# Supplementary material for: Programming bacteria for multiplexed DNA detection
Source: Nat Commun. 2023 Apr 10;14:2001. doi: 10.1038/s41467-023-37582-x (PMC10086068; doi:10.1038/s41467-023-37582-x)
Supplement: Supplementary file 2 — Reporting Summary [file 41467_2023_37582_MOESM2_ESM.pdf]

nature portfolio

Corresponding author(s): Ophelia S Venturelli

Last updated by author(s): March 22, 2023

Reporting Summary

Nature Portfolio wishes to improve the reproducibility of the work that we publish. This form provides structure for consistency and transparency in reporting. For further information on Nature Portfolio policies, see our Editorial Policies and the Editorial Policy Checklist.

Statistics

For all statistical analyses, confirm that the following items are present in the figure legend, table legend, main text, or Methods section.

n/a	Confirmed	
☐	☒	The exact sample size (*n*) for each experimental group/condition, given as a discrete number and unit of measurement
☐	☒	A statement on whether measurements were taken from distinct samples or whether the same sample was measured repeatedly
☐	☒	The statistical test(s) used AND whether they are one- or two-sided<br>*Only common tests should be described solely by name; describe more complex techniques in the Methods section.*
☒	☐	A description of all covariates tested
☒	☐	A description of any assumptions or corrections, such as tests of normality and adjustment for multiple comparisons
☐	☒	A full description of the statistical parameters including central tendency (e.g. means) or other basic estimates (e.g. regression coefficient) AND variation (e.g. standard deviation) or associated estimates of uncertainty (e.g. confidence intervals)
☐	☒	For null hypothesis testing, the test statistic (e.g. *F*, *t*, *r*) with confidence intervals, effect sizes, degrees of freedom and *P* value noted<br>*Give P values as exact values whenever suitable.*
☒	☐	For Bayesian analysis, information on the choice of priors and Markov chain Monte Carlo settings
☒	☐	For hierarchical and complex designs, identification of the appropriate level for tests and full reporting of outcomes
☒	☐	Estimates of effect sizes (e.g. Cohen's *d*, Pearson's *r*), indicating how they were calculated

Our web collection on statistics for biologists contains articles on many of the points above.

Software and code

Policy information about availability of computer code

Data collection	Microplate reader TECAN and its control software SPARKCONTROL (v2.3) were used to collect OD and fluorescence measurements. Nikon fluorescence microscope Eclipse Ti-E and its control software NIS-Elements AR (5.30.02) were used to collect bright-field and fluorescence images (GFP, RFP, and BFP) of bacterial colonies on agar plates. Azure Biosystem c300 was used to collect bright-field and fluorescence images (GFP only) of bacterial colonies on agar plates. Sequencing data were collected on an Illumina MiSeq instrument (MiSeq Control Software 3.1.0.13) and the BaseSpace Sequencing Hub's FASTQ Generation Program was used to generate FASTQ files for further analysis with custom Bioinformatics workflows.
Data analysis	Matlab R2019a (9.6.0.1072779) were used to generate plots of OD and fluorescence and perform unpaired unpaired t-test.<br>ImageJ (v1.53c) was used to process microscopic images for multiplexed detection. Custom Python scripts (run on Python 3) were used for 16S rRNA gene sequence analysis. Detailed method for 16S rRNA gene sequence analysis was described in the Method section.

For manuscripts utilizing custom algorithms or software that are central to the research but not yet described in published literature, software must be made available to editors and reviewers. We strongly encourage code deposition in a community repository (e.g. GitHub). See the Nature Portfolio guidelines for submitting code & software for further information.

Data

Policy information about availability of data

All manuscripts must include a data availability statement. This statement should provide the following information, where applicable:

- Accession codes, unique identifiers, or web links for publicly available datasets
- A description of any restrictions on data availability
- For clinical datasets or third party data, please ensure that the statement adheres to our policy

The source data for Figures 1-4 are including p-values are included and can also be downloaded from BioStudies database (https://www.ebi.ac.uk/biostudies/studies/S-BSST1044). Additional details such as plasmids, strains, and 16S rRNA gene analysis are available upon request to the corresponding author.

Human research participants

Policy information about studies involving human research participants and Sex and Gender in Research.

Reporting on sex and gender	N/A
Population characteristics	N/A
Recruitment	N/A
Ethics oversight	N/A

Note that full information on the approval of the study protocol must also be provided in the manuscript.

Field-specific reporting

Please select the one below that is the best fit for your research. If you are not sure, read the appropriate sections before making your selection.

[x] Life sciences [ ] Behavioural & social sciences [ ] Ecological, evolutionary & environmental sciences

For a reference copy of the document with all sections, see nature.com/documents/nr-reporting-summary-flat.pdf

Life sciences study design

All studies must disclose on these points even when the disclosure is negative.

Sample size	Sample size of 3 was chosen for all characterizations of the cell-based DNA sensors in biological replicates (3 independent transformations) or technical replicates (1 transformation with 3 plating). Transformation results were highly similar across either 3 biological replicates or 3 technical replicates. Sample size of 4 was chosen for plate reader (OD and fluorescence measurements) experiments specifically due to the observed variability. Unpaired, two-tailed Student t-test was performed by Matlab R2019a (9.6.0.1072779) to calculate p-values and determine if the detection time at a specific DNA concentration was different from the detection time of escape mutants based on the technical replicates (N=4). With sample size of 4, the detection times of cell-based DNA sensors were highly linear with the log transformed gDNA concentrations (Fig. 2d-g).
Data exclusions	No data were excluded from the characterizations of the cell-based DNA sensors.
Replication	Data presented in the paper had three to four biological or technical replicates performed in the same day. Experiments were performed at least twice by Yu-Yu Cheng or Zhengyi Chen independently. All attempts at replication were successful and highly reproducible.
Randomization	Randomization is not relevant to this study where samples were handled uniformly and the same data analysis was applied to all samples.
Blinding	Blinding is not relevant to this study where the cell-based DNA sensors were characterized by instruments and not subject to human biases. There was no experiments involved with human subjects research.

Reporting for specific materials, systems and methods

We require information from authors about some types of materials, experimental systems and methods used in many studies. Here, indicate whether each material, system or method listed is relevant to your study. If you are not sure if a list item applies to your research, read the appropriate section before selecting a response.

Materials & experimental systems

n/a	Involved in the study
x	Antibodies
x	Eukaryotic cell lines
x	Palaeontology and archaeology
	x Animals and other organisms
x	Clinical data
x	Dual use research of concern

Methods

n/a	Involved in the study
x	ChIP-seq
x	Flow cytometry
x	MRI-based neuroimaging

Animals and other research organisms

Policy information about studies involving animals; ARRIVE guidelines recommended for reporting animal research, and Sex and Gender in Research

Laboratory animals	8-week-old C57BL/6 gnotobiotic male mice (wild-type) were inoculated with 8 human gut bacteria - Dorea formicigenerans, Coprococcus comes, Anaerostipes caccae, Bifidobacterium longum, Bifidobacterium adolescentis, Bacteroides vulgatus, Bacteroides caccae, and Bacteroides thetaiotaomicron via oral gavage. After four weeks of colonization, mice were euthanized for cecal sample collection.
Wild animals	N/A
Reporting on sex	Sex of mouse was not relevant to the characterization of DNA sensors for the detection of spike-in bacteria in mouse ceca and was not considered in the experimental design.
Field-collected samples	N/A
Ethics oversight	Cecal samples were collected from germ-free mouse experiments following protocols approved by the University of Wisconsin-Madison Animal Care and Use Committee.

Note that full information on the approval of the study protocol must also be provided in the manuscript.
